# III-defined concepts in chemistry: rigid force constants *vs.* compliance constants as bond strength descriptors for the triple bond in diboryne

**DOI:** 10.1039/c5sc01322d

**Published:** 2015-05-04

**Authors:** Jörg Grunenberg

**Affiliations:** a Institut für Anorganische und Analytische Chemie , Technische Universität Braunschweig , Braunschweig 38106 , Germany . Email: Joerg.Grunenberg@tu-bs.de

## Abstract

In a recent publication, the interpretation of Braunschweig's diboryne NHC–BB–NHC as a true triple bond is questioned. The analysis by Köppe and Schnöckel is based, *inter alia*, on the calculation of rigid coupling force constants. Nevertheless, since it is known for a long time that the use of rigid force constants as bond strength descriptors is by no means straightforward, we recomputed the rigid force constants for a model diboryne, applying different coordinate systems and compared the values with the relaxed force constants (generalized compliance constants, GCC). In contrast with the results by Schnöckel and Köppe, the true coupling between the boron–boron bond and the boron–carbon bond, that is, after the elimination of all numerical artifacts, is negligible (*f*_BB/BC_ = –0.003).

## Abstract 2

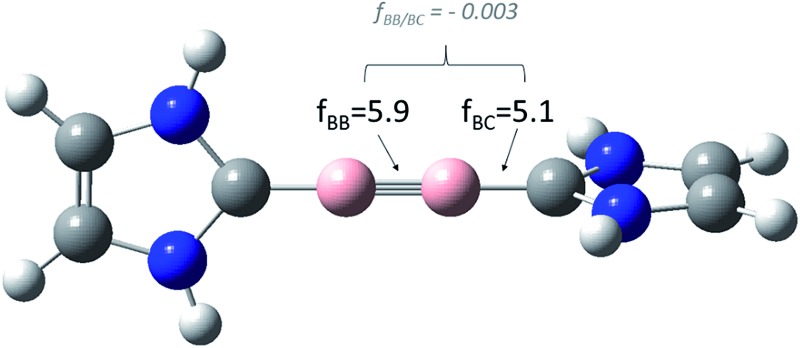
In a recent publication in this journal, the interpretation of the Braunschweig's diboryne as a true triple bond is questioned.

## Introduction

Computational chemistry has reached a high degree of maturity and comprehension making it one of the most active research areas in modern chemical and physical research in general. Predictions concerning single molecules, molecular clusters or even the solid state in combination with detailed information from apparatus based experiments are currently providing the ingredients to an auspicious revolution in the borderland between theory and experiment. Many, but not all, computational chemistry applications deal with observable properties. Here, one can always try to find an experiment, which allows either falsification or confirmation of the computer simulation. This is in sharp contrast to the second major application area of computational chemistry, the underpinning of chemical concepts, where a comparison with experiment is not always possible. Therefore, from time to time, even seemingly trivial questions frequently lead to highly active discussions in the scientific community. Especially the idea of bond orders and localized orbitals, are under debate.^1^

In a recent publication,^2^ the interpretation of the boron–boron triple bond in Holger Braunschweig's diboryne NHC–BB–NHC (NHC = N-heterocyclic carbene)^3^ as a triple bond is questioned. The analysis by Köppe and Schnöckel is based, *inter alia*, on the calculation of rigid coupling force constants. Nevertheless, since it is known for a long time that the use of rigid force constants as bond strength descriptors is by no means straightforward,^4^ we conducted the present study in order to evaluate the robustness of the numerical data.

## Computations

**Scheme 1:**
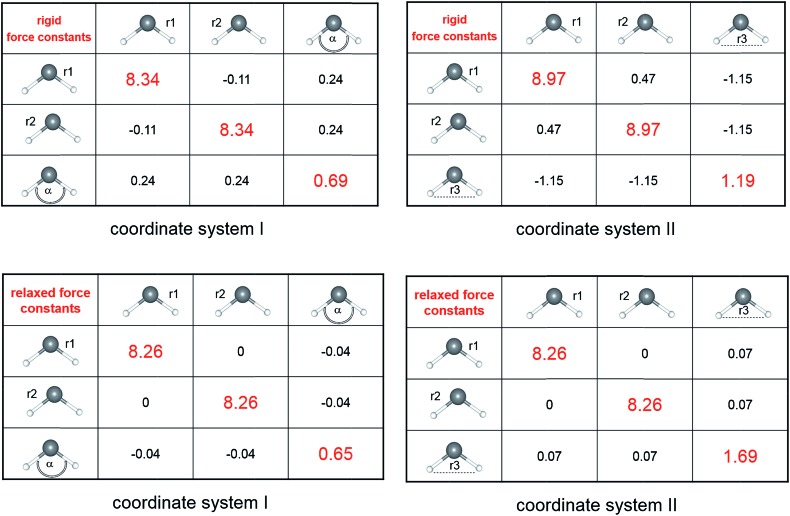
The matrices of *rigid* force constants (upper row) and *relaxed* force constants (lower row) for water computed at the CCSD(T)/aug-ccpv5z level of theory.

To analyze the numerical stability of *rigid* coupling force constants, as computed by Schnöckel und Köppe in ref. 2, we calculated, in a first step, the 3 × 3 matrix for H_2_O at the CCSD(T)/aug-cc-pvqz level of theory. Scheme 1 shows the matrix of force constants for water in terms of two different coordinate systems (**I** and **II**). Both systems have in common the two stretching coordinates OH(1) and OH(2). They differ only with respect to the third coordinate, which is the angle H–O–H or the H···H “stretching” coordinate, respectively. The *rigid* force constant matrix (coordinate system **I**) predicts a OH bond strength of 8.34 mdyn Å^–1^, while after transformation into coordinate system **II** the OH force constant significantly changes to 8.97 mdyn Å^–1^, simulating a stronger bond. Even more importantly, the stretch/stretch coupling constant does not only dramatically change its absolute value (from 0.11 to 0.47 mdyn Å^–1^), again simulating a strong electronic coupling, but also the algebraic sign from – to +. Any interpretation as a bond strength descriptor, is thus invalid. On the other side, looking at the *relaxed* force constant matrices (compliance matrix^5^) expressed in both coordinate systems **I** and **II**, a numerically stable sub-matrix can be identified. With 8.34 mdyn Å^–1^, the value of the relaxed force constant (inverse of the compliance constant) depicts a lower threshold of all possible coordinate systems. The same is true for the coupling constants. Both, value and sign, are no longer dependent on the coordinate system. In fact, the real OH/OH coupling constant vanishes completely.

Coming back to the diboryne question, the seemingly high BB/BC coupling force constant computed by Schnöckel and Köppe of +0.16 mdyn Å^–1^ piques one’s curiosity: is it “real” or is it an artifact of the coordinate system selected by the authors? This is important since, in the words of Schnöckel and Köppe “the interaction force constant means the interaction between two bonds, *i.e.* whether or not and to which extent there is a restoring force within the two bonds”.

As part of our ongoing project to develop *unique* numerical descriptors for chemical concepts, we introduced the method of generalized compliance constants (*GCC*) some years ago, as an extension to the theory of compliance matrices, valid for arbitrary non-stationary or stationary points on the potential energy hypersurface.^6^ The entries of the inverted Hessian matrix, the compliance constants, do not suffer from coordinate dependencies and can thus be assumed to be much more transferable between similar chemical environments.^7^

**Fig. 1:**
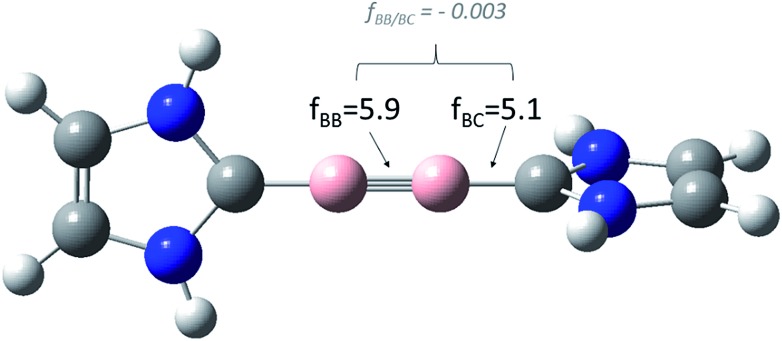
Relevant *relaxed* force and coupling constants, computed at the BP86/dz level of theory, applying the generalized compliance constants (*GCC*) approach,^6^ for the model system **1**. Both, the diagonal and the coupling terms are unique, that means, they do not depend on the definition of all other coordinates.

In order to separate real coupling phenomena from deceptive ones suggested by numerical artefacts, we – in a second step – therefore recomputed (1) the *rigid* force constants for Schnöckel's model system **1** of Braunschweig's diboryne, applying three different coordinate systems (see below) and (2) *relaxed* force constants applying our *GCC* formalism. In the following we denote the different coordinate systems according to Schnöckel's and Köppe's symmetry coordinates (S1); Peter Pulay's natural internal coordinates (S2); primitive internal z-matrix coordinates (S3) and finally our own generalized compliance coordinates,6*b* consisting of a redundant set of stretching coordinates (S4). For a better comparison with Schnöckel's data, all geometry optimizations and Cartesian force constants were computed at the BP87/dz level of theory. The transformation of the rigid Hessian matrices (S1, S2, S3) were done using Fogarasi's and Pulay's *fctint* code,^8^ while the relaxed force constants (S4) were computed using our COMPLIANC 3.0 code, freely available from our site ; www.oc.tu-bs.de/Grunenberg (Fig. 1).

As expected (see Table 1), our *relaxed* diagonal boron–boron (*f*_BB_ = 5.9 mdyn Å^–1^) and boron–carbon (*f*_BC_ = 5.1 mdyn Å^–1^) force constants comprise a lower boundary for all other possible coordinate systems. While the effect is quite small for Schnöckel's (S1) and Pulay's (S2) coordinate systems – both *f*_BB_ values are 6.0 mdyn Å^–1^ – the *f*_BB_ value expressed in *z*-matrix variables (S3) of 7.3 mdyn Å^–1^ again is “pretending” a stronger BB bond. The same is true for the boron–carbon bond.^9^ Most important nevertheless, the true coupling between the boron–boron bond and the boron–carbon bond, after the elimination of all numerical artifacts, is more or less negligible (*f*_BB/BC_ = –0.003).

**Table 1:** Relevant *relaxed* force and coupling constants in mdyn Å^–1^ (right column), computed at the BP86/dz level of theory, applying the generalized compliance constants (*GCC*) approach by Brandhorst and Grunenberg,^6^ as well as three different *rigid force constants* coordinate systems for the model system **1**

	(S1)	(S2)	(S3)	(S4)
*f*_BB_	6.0	6.0	7.3	**5.9**
*f*_BC_	5.2	5.2	5.4	**5.1**
*f*_BB/BC_	0.16	0.11	0.38	**–0.003** ^*a*^

^*a*^The unit of the coupling constant is given in Å mdyn^–1^.

In combination with a quick analysis of three model systems of archetypical B–B single, double and triple bonds and a comparison with the *relaxed force constant* of 6.3 mdyn Å^–1^ computed by Fischer and Braunschweig^10^ for the real diboryne (B_2_IDip_2_) allows a unique interpretation of the B–B bond under question as a triple bond.^11^
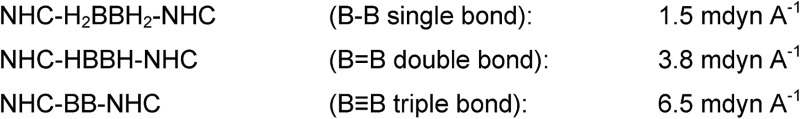


## Conclusions

(1) Rigid coupling force constants, as applied by Schnöckel and Köppe, are ill-defined and hence invalid as bond strength descriptors. The numerical values depend on the definition of all other coordinates. Relaxed force constants, on the other hand, do not depend on the coordinate system. They address the question “*which force has to be applied against a specific internal coordinate in order to achieve a given displacement, while all other forces thereby introduced are allowed to relax*”. The displacements of all other coordinates caused by these forces are given by the compliance coupling constants, which are the off-diagonal terms of the compliance matrix.

(2) If *rigid* force constants are employed for the description of individual bond strength anyhow, the bonding situation is always described as being too strong, since the values of rigid force constants are necessarily higher than the values of the corresponding relaxed force constants. This is of course also true for non-covalent interactions,^12^ even if there are still some misunderstandings on this aspect.^13^

(3) The true coupling between the boron–boron bond and the boron–carbon bond, after the elimination of all numerical artifacts, is negligible (*f*_BB/BC_ = –0.003).

Applying the method of generalized compliance constants (*GCC*), the calculation of *relaxed* force constants for covalent and non-covalent coordinates is now a straightforward task. It is somehow disturbing that, nearly 15 years after our original publication,^14^ the use of ill-defined, rigid force constants as bond strength descriptors is still prevalent.
